# Simulation of a computed HbA_1c_ using a weighted average glucose

**DOI:** 10.1186/s40064-016-1877-2

**Published:** 2016-02-29

**Authors:** W. Boutayeb, A. Boutayeb, M. Lamlili, S. Ben El Mostafa, N. Zitouni

**Affiliations:** URAC04, LaMSD, Department of Mathematics and Informatics, Faculty of Sciences, University Mohamed Ist, Oujda, Morocco; ISPITS Oujda Annex Nador, Health Ministry, Oujda, Morocco; Health Ministry, Oujda, Morocco

**Keywords:** Diabetes, Average glucose, Glycated haemoglobin, HbA_1c_, Weighted method

## Abstract

**Background:**

The A_1c_-derived average glucose examined the link between the glycated haemoglobin and the estimated average glucose, and provided a linear relation between them. Other studies proved that, over a period of 4 months, plasma glucose in the preceding 30 days contribute to about 50 % to the glycated haemoglobin value while the other 50 % is due to the remaining 3 months altogether.

**Technical details of the method:**

In this technical note, we propose a weighted method assuming that the contribution of glucose to glycated haemoglobin over 3 months is chronologically 20 %, 30 % and 50 % respectively. A comparison is made with the linear regression method which uses the same estimated average glucose over the whole period. Results yielded by the weighted method are also compared to those given by the model proposed by Ladyzyński et al.

**Findings:**

A simulation is carried out on data assumed to come from a first individual with nearly the same level of glucose over 3 months, a second individual who starts with high levels of glucose and then reaches a stabilised low level by the last month, and finally, a third case who had just been diagnosed with diabetes during the last month whereas he/she had a normal glycaemia during the preceding 2 months. The weighted method gives more realistic values of HbA_1c_ (7.36 %, 6.80 %, 8.49 %) than the linear regression method without weights which gives the same value (7.45 %) for the three cases. Another comparison shows that the three values given by the weighted method are slightly smaller than the corresponding values given by the model of Ladyzynski et al. (7.62 %, 7.02 %, 8.8 %) but the relative variation is nearly the same for the three values (≈3 %).

**Conclussion:**

Without regular self-testing and day-to-day insights, a sole HbA_1c_ value can be confusing and misleading. For physicians and patients, a clear understanding of the relationship between the weighted average glucose and HbA_1c_ is necessary in order to set an appropriate daily control depending on whether the glucose is stabilized over the whole period, at the beginning, at the end; or still under recurrent episodes of high and low levels. The measured HbA_1c_ at a biological laboratory gives no indication on glucose variation. Moreover, low values of glucose may cancel high values and lead to a “good” average glucose and ideal glycated haemoglobin.

## Background

The relationship between the glycated haemoglobin (HbA_1c_) levels and risks for diabetic complications in patients with type 1 (T1DM) and type 2 (T2DM) diabetes was established by the Diabetes Control and Complications Trial Research Group (DCCT) (1993, 1995) and the UK Prospective Diabetes Study Group (UKPDS) (1998a, b). More precisely, different epidemiologic studies and clinical trials have explored the relationship between HbA_1c_ and the average blood glucose (Hempe et al. 2002; Nathan 2009; Nathan et al. 2008; Barua et al. 2014). In particular, the A_1c_-derived average glucose (ADAG) examined the link between HbA_1c_ and the average glucose assessed as completely as possible with combinations of continuous glucose monitoring and frequent finger stick capillary glucose testing (Nathan 2009; Nathan et al. 2008). Using a linear regression analysis on data collected from 507 subjects, including T1DM, T2DM and non diabetic people from 11 centres in the US, Europe, Africa and Asia, the ADAG study provided a linear relation between HbA_1c_ and the estimated average glucose (eAG) (Nathan et al. 2008).

1 $${\text{eAG}}\left( {\text{mg/dl}} \right) \, = \, 28.7*{\text{eHbA}}_{{ 1 {\text{c}}}} \,\left( \% \right) - 46.7$$

Consequently, the HbA_1c_ assay became widely accepted and used for assessing chronic glycaemia (American Diabetes Association (ADA) 2015), and the quasi-totality of biological laboratories which carry out HbA_1c_ tests worldwide, indicate that a good control of diabetes requires an HbA_1c_ <7 % while an HbA_1c_ >10 % indicates a very bad control of diabetes.

During the last two decades, different mathematical models were proposed to deal with the relationship between HbA_1c_ and the average glucose (AG) (Temsch et al. 2008; Dayanand et al. 2012; Ladyzyński et al. 2008, 2011, 2014). Temsch et al. (2008) proposed a model respecting the decreasing contribution of older glucose levels to current HbA_1c_ values using truncated Fourier series and convolution. Dayanand et al. (2012) dealt with a comparison of calculated HbA_1c_ with measured HbA_1c_ by high Pressure Liquid Chromatography Method (Dayanand et al. 2012). Ladyzyński et al. (2008, 2011, 2014)published a series of papers on haemoglobin glycation rate constant and validation of haemoglobin glycation models. In the paper on haemoglobin glycation rate constant in non-diabetic individuals, they proposed and used the following mathematical model (Ladyzyński et al. 2011):

2 $$eHbA_{1c} (\% ) { } = \, 91.5 \, *\left( {1 - \frac{{1 - {\text{e}}^{{ - {\text{k}}\, \times \,{\text{LS}}\, \times \,{\text{eAG}}}} }}{{{\text{k}} \times {\text{LS}} \times {\text{eAG}}}}} \right) \, + \, 2.15,$$

where LS is the life span (=120 days), eAG is the average glucose supposed constant over the life span and *k* is the haemoglobin glycation rate constant.

In the paper “Validation of a haemoglobin A1c model in patients with type 1 and type 2 diabetes and its use to go beyond the average relationship of haemoglobin A1c and mean glucose level”, they used the previously cited model (Eq. 2) and carried out a linear regression of the data of the simulated ADAG population (i.e., with distribution of the eHbA_1c_ and eAG data identical with distribution of the data in the ADAG study) to obtain the following equation (Ladyzynski et al. 2014):

3 $$eHbA_{1c} (\%) = \, 0.0296 \, *{\text{ eAG (mg}}/{\text{dl) }} + \, 2.419$$

Rohlfing et al. (2002) used a linear regression on DCCT data, ending up with a relation similar to the one provided by ADAG, but referring to publications indicating that plasma glucose (PG) in the preceding 30 days contribute to about 50 % to the HbA_1c_ value and PG from 90 to 120 days earlier contribute only to about 10 % (Rohlfing et al. 2002). More precisely, Tahara and Shima (1993) suggested that 50 % of HbA_1c_ is determined by the PG level during the preceding one month period and that 25 % of HbA_1c_ is determined by the PG during a prior 1 month period, the remaining 25 % of HbA_1c_ level is therefore determined by the PG level during the 2 months period before these 2 months (Tahara and Shima 1993). In a response to Trevino who proposed a mathematical formula for HbA_1c_ change in response to exponential PG decay (Trevino 2006), Tahara considered a slightly different relationship between PG and HbA_1c_. “For T = 120 days, 50 % of A1C is determined by the PG level during the preceding 35 days, 25 % by the PG level during 25 days before this period and the remaining 25 % by the PG level during the 2 months period before these periods” (Boutayeb and Lamlili 2015).

In a recent paper, we showed that low values of HbA_1c_ may be dangerous under recurrent episodes of hypoglycaemia (Trevino 2006). In the present technical note, we propose a simulation of a “weighted” version of Eq. 3 proposed by Ladyzynski et al. (2014), using a corrected average glucose (wAG) over 3 months instead of the uniform eAG. Based on the relationship between PG and HbA_1c_ given by Tahara and cited earlier, we assume that, over 3 months, the earliest, middle and recent month contribution of glucose to wHbA_1c_ is 20, 30 and 50 % respectively. The limitation to a period of 3 months instead of four is rather a pragmatic choice linked to the blood glucose measurement by diabetic patients. In fact, the simulation with 4 months with weights: 10, 15, 25 and 50 % yields nearly the same results as with 3 months.

## Technical details of the method

Considering Eq. 3 given in the introduction section, we compute an HbA_1c_ using a “weighted” average glucose (wAG) as follows:

4 $${\text{wAG }} = \, 20\,\% *{\text{AGM}}_{ - 3} + \, 30\,\% *{\text{AGM}}_{ - 2} + \, 50\,\% *{\text{AGM}}_{ - 1} \,{\text{and } wHbA_{1c} } = \, 0.0296*{\text{wAG }} + 2.419$$

where AGM_−1_, AGM_−2_ and AGM_−3_ represent the average glucose computed chronologically over the preceding months to the wHbA_1c_ test.

To see the effect of weighted average glucose, we compare the computed HbA_1c_ obtained by using the formulas (Eq. 3) and (Eq. 4) on the basis of simulated data assumed to belong to three individuals having the same 3 months mean blood glucose level (170 mg/dl):

1. Individual with Steady State Control (ISSC) having an average glucose (mg/dl) nearly the same over the 3 months period (AGM_−3_ = 180, AGM_−2_ = 170, AGM_−1_ = 160),
2. Individual with Improving Control (IIC) with an average glucose (AGM_−3_ = 250, AGM_−2_ = 160 in mg/dl) high at the beginning of the 3 months period and normal at the most recent month (AGM_−1_ = 100 mg/dl),
3. Individual Newly Diagnosed (IND) starting with a normal average glucose (AGM_−3_ = 100, AGM_−2_ = 100 mg/dl) at the 1st and 2nd months and reaching a high level at the most recent month (AGM_−1_ = 310 mg/dl).

We are aware that using data of real patients would be more interesting than simulation of hypothetical data of three patients. To overcome this shortage, we compare our results with those yielded by the model proposed by Ladynzinski et al. (Eq. 2), with k = 1.296 × 10^−9^ l/(mmol * s).

## Results

Using Eq. 3 and the “weighted” formula given by Eq. 4, we obtain the results given in Table 1.

**Table 1:** A comparison of HbA_1c_ obtained by using Eq. 3 and the weighted method (Eq. 4) and also a comparison of results given by Eq. 2 to those given by Eq. 4

	Average glucose/month (mg/dl)			Average glucose for 3 months	Computed average glucose/month (mg/dl)		Computed HbA_1c_ (%)		Computed HbA_1c_ (%) using Ladyzinski et al. model (Eq. 2)	
	AGM_−3_	AGM_−2_	AGM_−1_		eAG using (Eq. 3)	wAG using (Eq. 4)	eHbA_1c_ using (Eq. 3)	wHbA_1c_ using (Eq. 4)	eHbA_1c_ using (Eq. 2)	wHbA_1c_ using (Eq. 2)
ISSC	180	170	160	170	170	167	7.45	7.36	7.71	7.62
IIC	250	160	100	170	170	148	7.45	6.80	7.71	7.02
IND	100	100	310	170	170	205	7.45	8.49	7.71	8.80

AGM_-1_, AGM_-2_, AGM_-3_ represent the average glucose for the 1st, 2nd and 3rd month preceding the HbA_1c_ test and average glucose for 3 months is calculated as (AGM_-1_ + AGM_-2_ + AGM_-3_)/3

*ISSC* individual with steady state control, *IIC* individual with improving control, *IND* individual newly diagnosed

## Conclusion

A comparison between the two methods used to compute HbA_1c_ shows that for the first individual, the two values (7.45 % vs 7.36 %) are relatively close to each other, compared with the values of HbA_1c_ obtained for the second individual (7.45 % vs 6.80 %) and the third one (7.45 % vs 8.49 %).

Contrarily to the first method (Eq. 3) which gives the same value of HbA_1c_ (7.45 %) for the three individuals, the results provided by the second method (Eq. 4) are more realistic since the first individual appears to have stabilised his/her glucose over the whole 3 months period. The second individual seems to have started with a high level of glucose but he/she has stabilised it during the most recent month. In opposition, the third case seems to have just been diagnosed with diabetes during the most recent month.

Comparing the results yielded by the proposed weighted method to those given by the model of Ladyzinski et al. indicates that the three values of wHbA_1c_ (7.36 %, 6.8 % and 8.49 %) given by Eq. 4 are slightly smaller than the corresponding values given by Eq. 2 (7.62 %, 7.02 % and 8.8 %) but the relative variation is nearly the same for the three values (≈3 %). It should be stressed that the model of Ladyzinski et al. (Eq. 2) was obtained by integrating the differential equation dHbA_1c_(t)/dt= −kHbA_1c_(t)AG(t), under the assumption of a constant glycemia throughout the entire life span of erythrocytes.

The “weighted” method indicates clearly that, for physicians and patients, a clear understanding of the relationship between wAG and wHbA_1c_ is necessary in order to set an appropriate daily control depending on whether the glucose is stabilised over the whole period, at the beginning, at the end; or still under recurrent episodes of high and low levels. The measured HbA_1c_ at a biological laboratory gives no indication on glucose variation. Moreover, low values of glucose may cancel high values and lead to a “good” average glucose and ideal glycated haemoglobin. Our simulation indicates that weighted HbA_1c_ could help patients with diabetes to better control their blood glucose by going beyond the simple average and trying to detect the variability of glucose during the whole period.
